# Predicting the response of olfactory sensory neurons to odor mixtures from single odor response

**DOI:** 10.1038/srep24091

**Published:** 2016-04-07

**Authors:** Addolorata Marasco, Alessandro De Paris, Michele Migliore

**Affiliations:** 1 University of Naples Federico II, Department of Mathematics and Applications, Naples, 80126, Italy; 2 Yale University School of Medicine, Department of Neurobiology, New Haven, 06520, USA; 3 National Research Council, Institute of Biophysics, Palermo, 90146, Italy

## Abstract

The response of olfactory receptor neurons to odor mixtures is not well understood. Here, using experimental constraints, we investigate the mathematical structure of the odor response space and its consequences. The analysis suggests that the odor response space is 3-dimensional, and predicts that the dose-response curve of an odor receptor can be obtained, in most cases, from three primary components with specific properties. This opens the way to an objective procedure to obtain specific olfactory receptor responses by manipulating mixtures in a mathematically predictable manner. This result is general and applies, independently of the number of odor components, to any olfactory sensory neuron type with a response curve that can be represented as a sigmoidal function of the odor concentration.

The possibility to express the response of olfactory sensory neurons (OSNs) to odor stimuli, in terms of a few elementary and objective responses, would be an extremely useful step to understand, and eventually create, specific perceptual representations. Classically, one can say odors can be composed using combinations of response magnitudes of all the receptors but, surprisingly, so far it has not been possible to identify clear primary components, in spite of widespread and intense multidisciplinary experimental (reviewed in ref. 1), theoretical^2^, and computational^3^ efforts. These would be a (small) set of specific dose-response curves from which can be constructed the corresponding curve of any OSN targeting any given glomerulus in the presence of any odor in any type of mixture. The large number of different OSNs, each often expressing a single receptor gene, and the consequent combinatorial explosion of the possible odor responses, has been considered as an implicit proof that “primary odor responses” do not exist. Their identification, however, would be highly significant, from increasing our understanding of odor coding and perception to practical and industrial applications. For this reason, in this paper we focus our attention on the responses elicited in OSNs by odor inputs, in the attempt to find what we call primary odor responses. We first find an appropriate formulation to take into account the response obtained when different odors are combined into a mixture, and then explore its mathematical structure to identify primary components. The analysis suggests that the odor response space is 3-dimensional, and predicts that the dose-response curve of any odor receptor can be obtained from three (out of four) primary components with specific properties.

## Results

**Figure 1:**
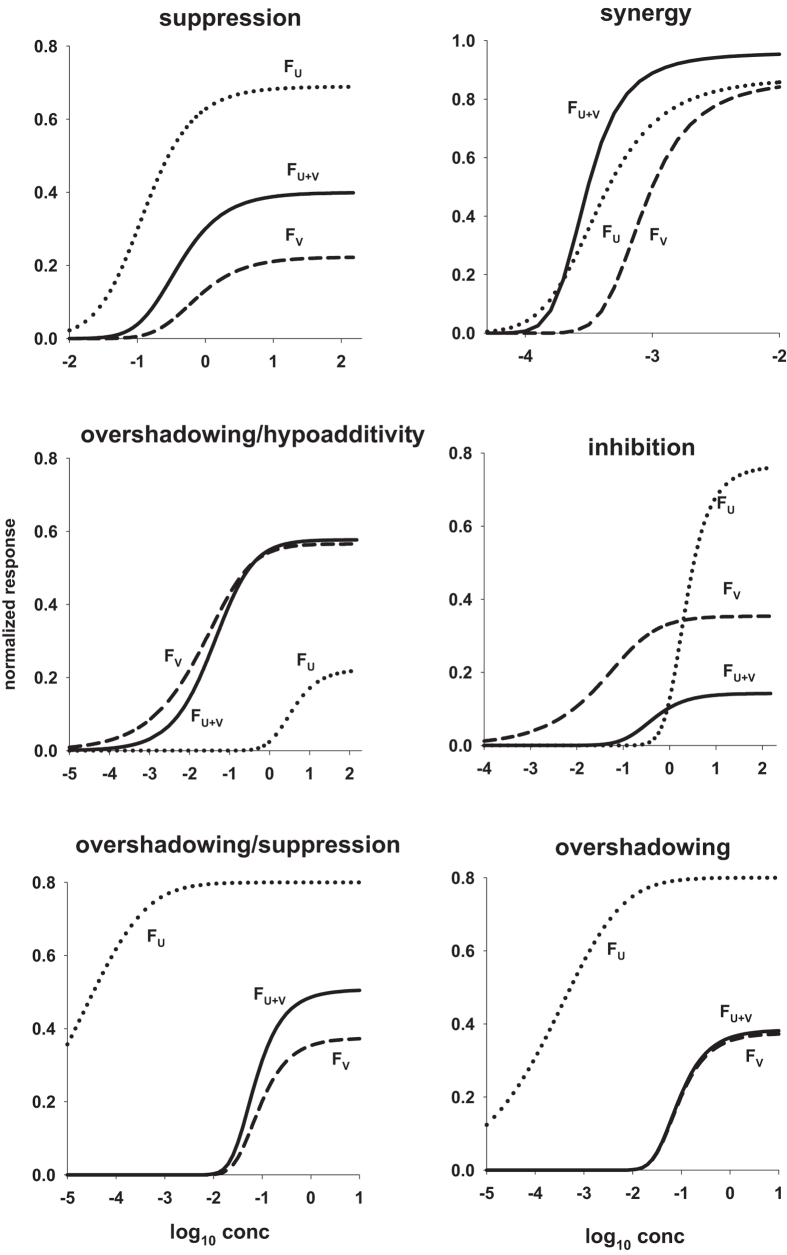
The model can reproduce all types of odor response to mixtures. In each panel is reported a typical set of odor response to single odors (*F*_*U*_ and *F*_*V*_) and their binary mixture (*F*_*U*+*V*_). Unless otherwise noted odors were mixed at a fixed concentration ratio of *r* = 0.2. Eqs. (3) and (5) were used with the following parameters for each panel; *suppression: n*_*U*_ = 1.5, *n*_*V*_ = 3.5, *η*_*U*_ = 1.7, *η*_*V*_ = 0.7, *K*_*U*_ = 0.2, *K*_*V*_ = 0.2; *synergy: n*_*U*_ = 3.6, *n*_*V*_ = 19.6, *η*_*U*_ = 1.7, *η*_*V*_ = 1.1, *K*_*U*_ = 3.16 ⋅ 10^−4^, *K*_*V*_ = 10^−4^, *r* = 1; *overshadowing/hypoadditivity: n*_*U*_ = 3.5, *n*_*V*_ = 0.5, *η*_*U*_ = 0.7, *η*_*V*_ = 1.7, *K*_*U*_ = 0.2, *K*_*V*_ = 0.2; *inhibition: n*_*U*_ = 4.5, *n*_*V*_ = 0.5, *η*_*U*_ = 1.3, *η*_*V*_ = 0.3, *K*_*U*_ = 0.2, *K*_*V*_ = 0.2; *overshadowing/suppression: n*_*U*_ = 0.5, *n*_*V*_ = 10, *η*_*U*_ = 16, *η*_*V*_ = 0.95, *K*_*U*_ = 0.51 ⋅ 10^−3^, *K*_*V*_ = 0.9 ⋅ 10^−2^, *r* = 1.6; *overshadowing: n*_*U*_ = 0.5, *n*_*V*_ = 10, *η*_*U*_ = 16, *η*_*V*_ = 0.95, *K*_*U*_ = 0.8 ⋅ 10^−2^, *K*_*V*_ = 0.9 ⋅ 10^−2^, *r* = 2 ⋅ 10^−4^.

We begin by considering the experimental findings on the activation of the olfactory sensory neurons (OSNs) as a function of an odor concentration, i.e. a dose-response curve for a specific OSN type. It has been widely demonstrated that in most cases this response can be conveniently reproduced by a sigmoidal shape with concentration, usually implemented as a Hill function. In the following, we will consider only responses that can be reproduced in this way. The general functional form for the response to an odorant *U, F*_*U*_, is thus


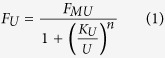


where *U* is also the concentration of the odorant, *n* is the Hill coefficient, and *K*_*U*_ and *F*_*MU*_ depend on the receptor kinetic properties^4^,^5^. From a mathematical point of view, *n* modulates the steepness of the curve, *K*_*U*_ the midpoint, and *F*_*MU*_ the asymptotic value. This function has been used to successfully fit the great majority of OSN responses that have been experimentally reported so far for single odors. However, it needs to be generalized for mixtures, since the OSN response to a combination of odors cannot be obtained from the simple sum of the functions for the individual components. Experimentally, the dose-response curve for a binary odor mixture at a fixed concentration ratio is still of sigmoidal shape in most cases, and it is usually compared with the curves for the individual components by using conventional types of behavior that can be exhibited for a more or less wide range of concentration^5^,^6^. One of the possible classifications is based on the following empirical definitions (illustrated in Fig. 1): *i) suppression*, when the response to the mixture is between the response to the single components, *ii) hypoadditivity*, when the response to the mixture is similar to the most effective component, *iii) synergy*, when the response to the mixture is higher than both components, *iv) inhibition*, when the response to the mixture is lower than both components, *v) overshadowing*, when the response to the mixture is similar to one of the components.

To take into account mixtures, it has been proposed^4^,^5^ to extend the formula for the response to a single odor as


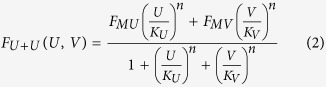


where *U* and *V* are two odors (and also their concentrations) and the other parameters are those defined for the single odor case. This approach is able to explain all cases of suppression but it can be mathematically demonstrated (see Appendix A.1) that it cannot reproduce mixtures exhibiting synergy, and inhibition, at least in the region of the maximum response. It has also been noted^7^ that it cannot take into account the self-mixture case (i.e. it should be *F*_*U*+*U*_(*U, U*) = *F*_*U*+*U*_(2*U*, 0)). In a somewhat different implementation^7^ it was proposed to use, for single odors and binary mixture


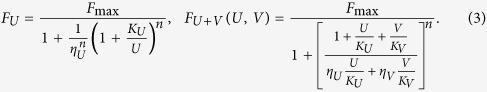


Adding the *η* parameter was a nice way to use the same *F*_max_ for all odors while allowing different asymptotes. This is equivalent to assume that, although the maximum signal generated by an OSN can be limited (up to *F*_max_) by the intrinsic properties of these type of cells, its maximal response to different odors can still be different for a variety of biochemical reasons that are implicitly taken into account by *η*. Although this model is consistent with the self-mixture case, it can also be mathematically demonstrated that it has the same limitations of that proposed in^5^ (see Appendix A.1).

The major problem with these formulations is that the same Hill coefficient, *n*, describing cooperative effects in the transduction cascade, is used for all types of OSNs. It has been assumed that this coefficient is a property of the neurons instead of the odors. However, there are experimental findings^8^ showing that the response of the same OSN to different odorants may exhibit different Hill coefficients, even in response to structurally similar odorants. We thus propose to describe the dose response curves for a given type of OSNs in the presence of odors *U* and *V* as


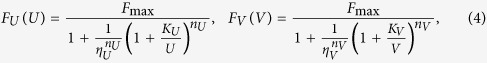


and their mixture by


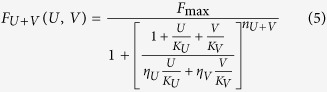


where we assumed *F*_max_ to be the same for all OSNs and related to the maximal physiological response that can be produced, and


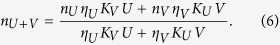


In Appendix A.2 are reported more details and the generalization to mixtures of *N* odorants. Note that if *U* = *rV*, that is for a mixture with a fixed concentration ratio, the dose-response curve will still be of sigmoidal shape


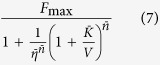


where


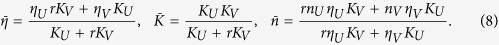


If *n_U_* = *n_V_* the equations above reduce to those proposed in^7^.

It is important to stress that all types of interaction between odors in a mixture can be taken into account by this formulation. Typical examples are reported in Fig. 1, where we plot six different cases. All the typical, experimentally measurable, characteristics of the curve for the mixture (i.e. steepness, midpoint, and asymptotic value) can be mathematically expressed by a non-linear combination of the parameters for the individual components (see Appendix A.2). Using our model, it is thus possible to find suitable mathematical conditions for all parameters and concentrations (see Appendix A.3) to design mixtures with specific properties.

**Figure 2:**
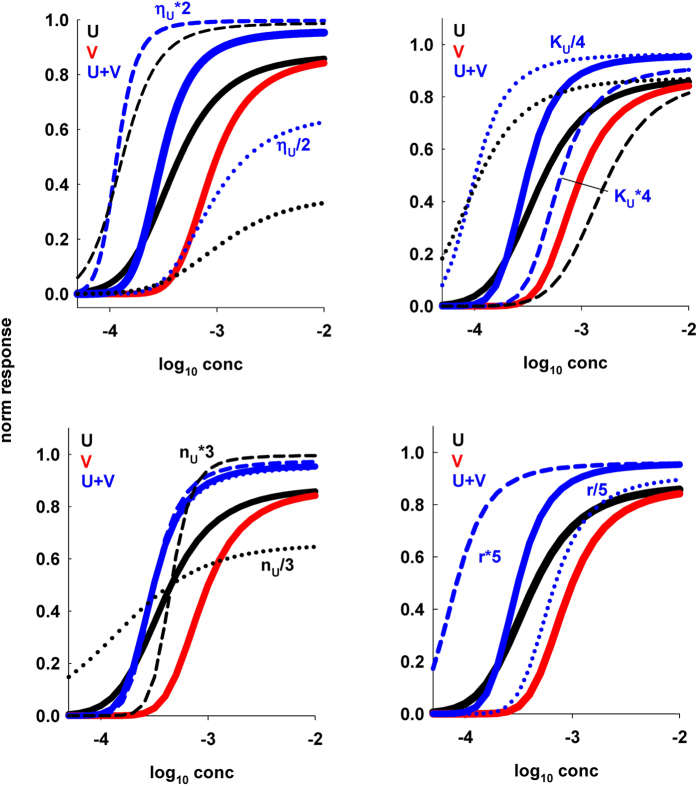
Changes in one of the odor parameters can significantly alter the mixture behavior. In all panels are plotted the same control curves for two odors (black and red solid lines for odor *U* and *V*, respectively) and their mixture (blue solid line, Eq. (5)) with parameters: *n*_*U*_ = 3.6, *n*_*V*_ = 19.6, *η*_*U*_ = 1.7, *η*_*V*_ = 1.1, *K*_*U*_ = 3.16 ⋅ 10^−4^, *K*_*V*_ = 10^−4^, *r* = 1; in each panel, one of the parameters for the response to odor *U* (black lines) is changed as indicated; dotted and dashed black lines indicate the change in the response to odor *U*, dotted and dashed blue lines indicate the change in the mixture. Note that a change in the concentration ratio, r, does not affect the single odor curves.

To see how the shape of the curve for a mixture is affected by the properties of the individual components, in Fig. 2 we report the results obtained by changing, one at the time, the parameters for one of the odors, *n*_*U*_, *η*_*U*_, *K*_*U*_, and the concentration ratio for the mixture, *r*. In all cases we started from single odors that result in a mixture exhibiting suppression or inhibition at low concentration, and synergy for higher concentration (thick black, red, and blue lines in Fig. 2). Changes in either *η*_*U*_, or *K*_*U*_ (Fig. 2, top panels) can change the range of concentration for which the mixture exhibits synergy, whereas only small effects can be observed by changing the Hill coefficient, *n*_*U*_. Much bigger effects are observed by changing the mixture concentration ratio (Fig. 2, bottom right panel); an increase or decrease of *r* can result in a substantial change of the mixture behavior, and a consequent change in the width of the concentration range exhibiting synergy or suppression.

**Figure 3:**
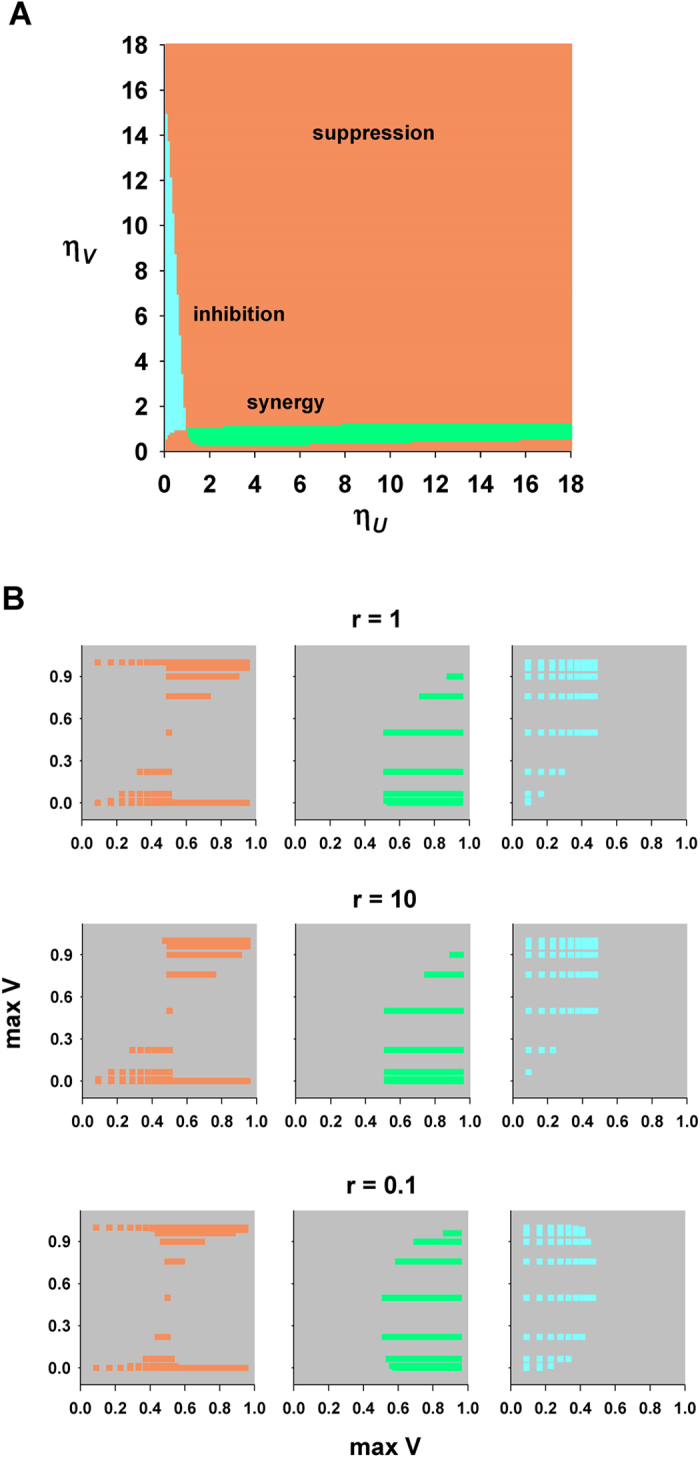
The model can predict the properties of an odor mixture from the single odor parameters. (**A**) The plot shows the property of a binary mixture of odors *U* and *V* as a function of *η*_*U*_ and *η*_*V*_ (with step 0.1). All other parameters were fixed as follows: *n*_*U*_ = 1, *n*_*V*_ = 12, *K*_*U*_ = 0.5 ⋅ 10^−4^, *K*_*V*_ = 10^−3^, *r* = 1; different basic mixture properties, as defined in the main text and App.A.1, are indicated using different colors: orange, suppression; cyan, inhibition; green, synergy; all properties were classified by analyzing the portion of the curve close to the asymptotic value; (**B**) asymptotic values, max *U* and max *V*, calculated according to Eq. (17) in App.A.2, using the set of *η*_*U*_ and *η*_*V*_ as in part A and different values of concentration ratio, *r*. The gray background highlights asymptotic values that cannot be obtained with the set of parameters used for panel A (with step 0.1 for *η*_*U*_ and *η*_*V*_).

Another, different, view of how single odor parameters can affect the properties of their mixture is shown in Fig. 3. Using the asymptotic value of the response as a reference for mixture classification, in Fig. 3A we show the result of mixing two odors with different *η*. As can be seen, most combinations would result in suppression, and only a relatively small fraction in synergy or inhibition. For experimental investigations, it can be more practical to use the asymptotic response obtained for the single odors to predict the mixture behavior. In Fig. 3B we thus plotted the data used for Fig. 3A (with *r* = 1) as a function of the asymptotic response to odors *U* and *V*, for different values of the odor concentration ratio (i.e. *U* = *rV*, with *r* = 0.1, 1, 10). The results suggest experimentally testable predictions on the relation that should exist between the peak response to odor *U* and that for odor *V* and their concentration ratio. Note how lowering the concentration ratio can substantially change the mixture behavior. Taken together these results thus suggest a new way to predict the overall behavior of a receptor type in response to a mixture composed by any number of components, provided that the individual response curves and their relative concentration ratio are known.

Given the rather intricate relation between the parameters for single odor and those for their mixture, it can be expected that to have a reasonable validation of the model against experimental data it is very important to have an exact description of the experimental protocol used to study the mixture, and as much as possible information on the response for the individual components. Unfortunately, these types of experiment appear to be technically challenging and with an intrinsically high physiological variability. It is thus not always possible to gather enough information to completely characterize the responses, and in particular their asymptotic value.

To test our model we used as reference the findings obtained in three studies, using different experimental protocols: 1) the work by Rospars *et al*.^5^, discussing typical examples of single odors and binary mixtures in which responses was measured using the same dilution in all cases and plotted as a function of the single odor and mixture concentration; 2) the work by Cruz and Lowe^7^, in which experimental data points were plotted as a function of the concentration of one odor with a second odor added at a fixed concentration, 3) the work by Duchamp-Viret *et al*.^6^, using the same protocol as in Rospars *et al*.^5^. The results were classified in Appendix A.4 using both the mean square error (*MSE*) and the mean absolute percentage error (*MAPE*), reported in Supplementary Tables S2–S3 for all curves. In almost all cases, we found a very good qualitative agreement with experimental data.

**Figure 4:**
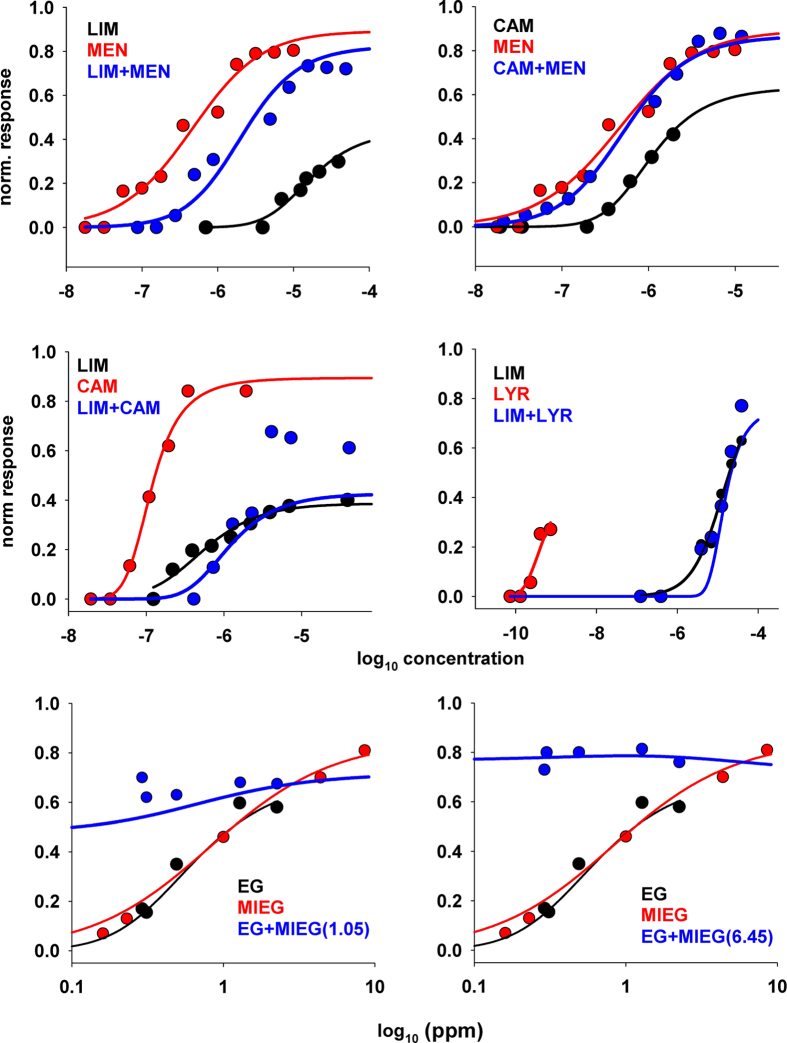
Model validation against experimental findings. In each panel are reported the experimental responses recorded in an OSN to two single odors (black and red symbols) and to their mixture at the same dilution (blue symbols) as a function of the concentration. Black and red lines represent best fits of experimental points, according to Eq. (3). The blue line in each panel represents the model prediction for the mixture, using Eqs. (25) and (27) in App.A.3, with the parameters found for single odors. Experimental points for LIM, MEN, CAM, LYR, and their mixture provided by JP Rospars (personal communication); experimental points for EG, MIEG, and their mixture taken and redrawn from Fig. 3C,D of ref. 7.

For the data from Rospars *et al*.^5^, using Eq. (4) we first fitted the experimental data (kindly provided by JP Rospars, together with precise information on the experimental protocol) on the response to single odors (LIM, CAM, MEN, and LYR, red and black lines and symbols in Fig. 4) to obtain an estimate of the parameters *n, η*, and *K* in each case. Next, using these parameters and the molarity of saturating vapor of each component (reported in Table 1 of ref. 5 for each odor component), we predicted the dose-response curve for the mixture using Eq. (7) adapted to take into account the specific experimental protocol used in these experiments (see App.A.3, Eq. (25)). Six cases are shown in Fig. 4. The top four panels demonstrate that the model is able to correctly reproduce suppression (Fig. 4, top left), hypoadditivity (Fig. 4, top right), and inhibition (Fig. 4, middle panels). For the other set of experiments, data points were taken and redrawn from Fig. 3C,D of the ref. 7. In this case, as stated above, experimental points were explicitly plotted as a function of the concentration of one odor (EG) with a second odor added at a fixed concentration (MIEG at 1.05 or 6.45 ppm). The Eq. (5) is used to predict the mixture for this experimental protocol (see App. A.3 Eq. (27)). As can be seen from the bottom panels of Fig. 4, also in this case the model was in very good agreement with experiments over the entire concentration range that was tested. Taken together, these results suggest that the model is able to qualitatively predict all the types of mixture behavior that are observed experimentally.

**Figure 5:**
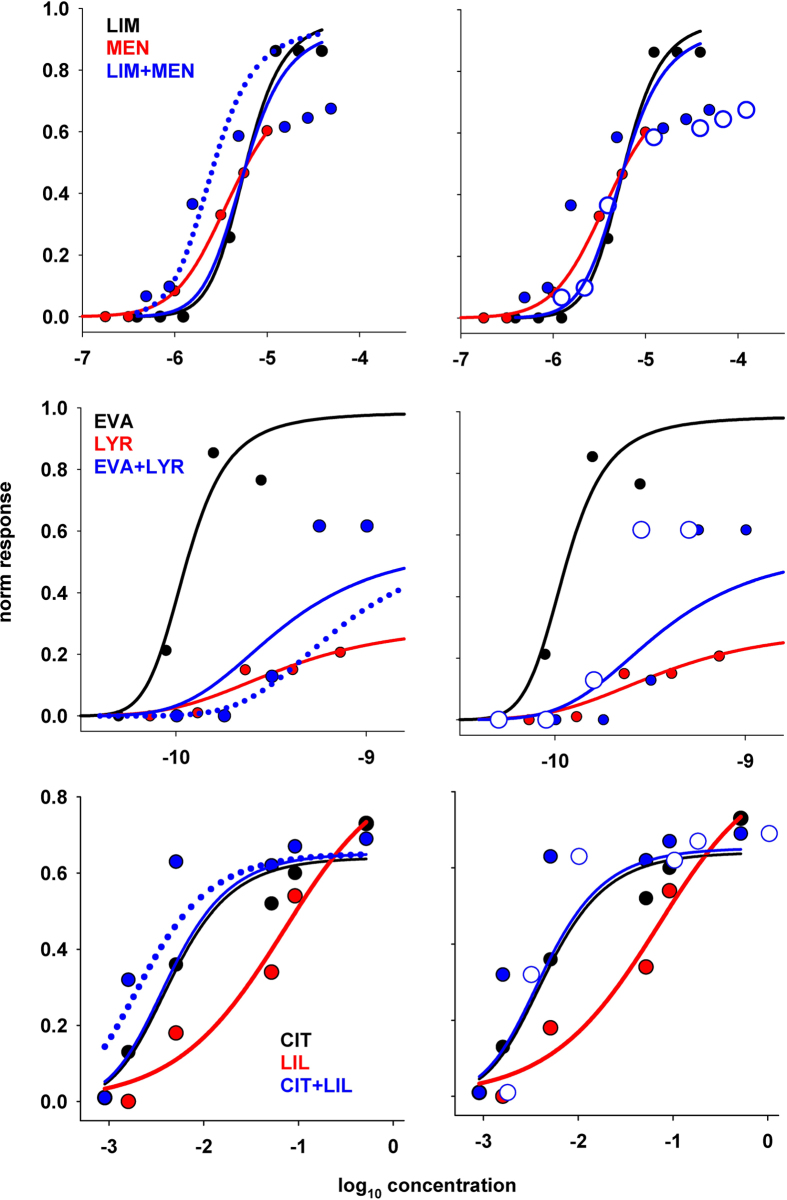
Cases in which the model failed to reproduce experimental findings. Model validation against experimental findings. In each panel are reported the experimental responses recorded in response to two single odors (black and red symbols) and to their mixture (blue symbols) as a function of the concentration. Black and red lines represent best fits of experimental points, according to Eq. (3); the solid blue line in each panel represents the model prediction for the mixture, using Eq. (25) in App.A.3, with the parameters found for single odors; Dotted blue lines represent the model prediction shifted by −0.06, +0.026, and −0.1 log_10_(*conc*) for LIM + MEN, EVA + LYR, and CIT + LIL, respectively; open blue symbols represent a shift in the experimental points for the mixture. Experimental points for LIM, MEN, EVA, LYR, and their mixture provided by JP Rospars (personal communication); experimental points for CIT, LIL, and their mixture taken and redrawn from Fig. 3 of ref. 6.

Three cases in which the model failed to reproduce the experimental findings are shown in Fig. 5, and refer to experiments exhibiting synergy (LIM + MEN, and CIT + LIL) and inhibition (EVA + LYR). We first tried to obtain different fits of the single odor responses using different initial constraints for the parameters, but the problem persisted (not shown). In order to figure out the possible reasons for this discrepancy we note that, with our model and under the experimental paradigm of Rospars *et al*.^5^ and Duchamp-Viret *et al*.^6^, it can be mathematically demonstrated that synergy cannot be obtained for concentration values below the midpoint of the component with the lower Hill coefficient (see App.A.3). The problem in reproducing these data may just be an intrinsic model limitation. However, there can be another reason, related to the way in which the results for the mixture are determined and represented; we noted that in all cases a simple shift in the concentration assumed to plot the result for the mixture models (−0.06, +0.026, and −0.1 log_10_(*conc*) for LIM + MEN, EVA + LYR, and CIT + LIL, respectively) would result in a much better agreement with experiments, especially in the lower concentration range (dotted line in left panels of Fig. 5). This is equivalent to suggest that the real mixture concentration in these cases may have been different from that expected or calculated from SVP. In these cases, the experimental points for the mixture (Fig. 5, blue symbols in left panels) should thus be shifted accordingly, as shown in the right panels of Fig. 5 (open blue symbols in right panels). The overall result would thus be overshadowing for LIM + MEN and CIT + LIL or suppression for EVA + LYR. In Supplementary Table S3 *MSE* and *MAPE* are also reported for the mixture curve corrected as explained. These findings suggest that, if the model holds true, errors in the calculation of the mixture concentration can result in a significant change in the interpretation of a mixture’s behavior.

With the formulation defined by Eqs. (3) and (7), able to take into account all the experimentally observed odor responses and interactions (as demonstrated in Figs. 1, 2 and 4), we can carry out a rigorous analysis of the mathematical structure of the odor response space and its consequences. Let us consider the function


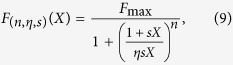


where the variable *X* is a real positive number, and the parameters *n, η*, and *s* are all positive.

Note that when *s* = 1/*K* and *X* is an odorant concentration we get Eqs. (7) and (3). In this way, any dose-response curve for a given type of OSNs can be univocally identified by the triple (*n, η, s*), and the whole odor response space (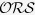) will be


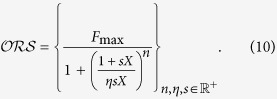


It should be stressed that this formulation is valid only for those responses that can be represented with a Hill function (i.e. with a sigmoidal shape as a function of the concentration). Next, we need to represent (mathematically) the response to an arbitrary combination of these curves. This is analogous to find/define the rules to compose vectors in space. It is a fundamental step since, as mentioned above, it is easy to figure out that the response to the mixture cannot be simply calculated from the algebraic sum of the responses to the individual components. Several theoretical composition laws could have been envisioned. We choose one of the simplest heuristic generalizations that reduce to the Cruz and Lowe model when using the same Hill coefficient for both components. We represent it with the following two operations:


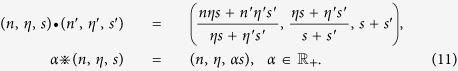


where 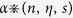 identifies the response to odorant (*n, η, s*) at concentration *αX*, i.e.


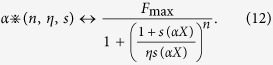


and (*n, η, s*)•(*n*′, *η*′, *s*′) identifies the response to a mixture of two odorants (*n, η, s*) and (*n*′, *η*′, *s*′), i.e.


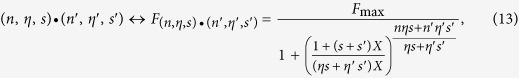


Note the special symbols • and 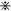 that we decided to use to define these operations. This is needed to stress that the operation they carry on the operands are different from the usual algebraic operations. Also note that to any combination of (*n, η, s*) corresponds a unique combination for (*nηs, ηs, s*). Mathematically, the space constructed in this way is a 3–dimensional domain that embeds into the standard topological vector space 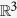 by means of the map


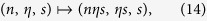


from which we obtain that


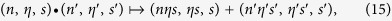



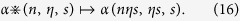


An important feature of the vector space structure of 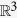 is that the choice of three independent vectors (i.e. a basis) suffices to generate the whole space. The map defined by Eq. (14) could then be used to carry a similar feature on the odor space. Thus, under the appropriate constraints, any odor response can be represented by a combination of a basis formed by three primary responses, i.e.,


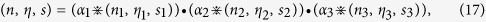


where


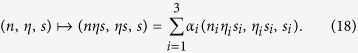


Because only positive values of *n*, and *η* are physiologically relevant, the odor space will be mapped only on the positive octant 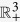. Thus, consider three vectors in 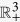 (which corresponds to three arbitrary odor responses)


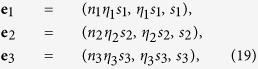


then they can constitute a basis only if


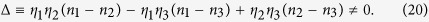


Under this condition, any triple (*nηs, ηs, s*) is uniquely obtained as a combination of **e**_1_, **e**_2_ and **e**_3_, i.e.


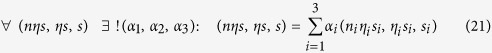


The scalars in the equation above must be positive and can be explicitly determined as


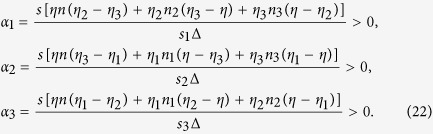


**Figure 6:**
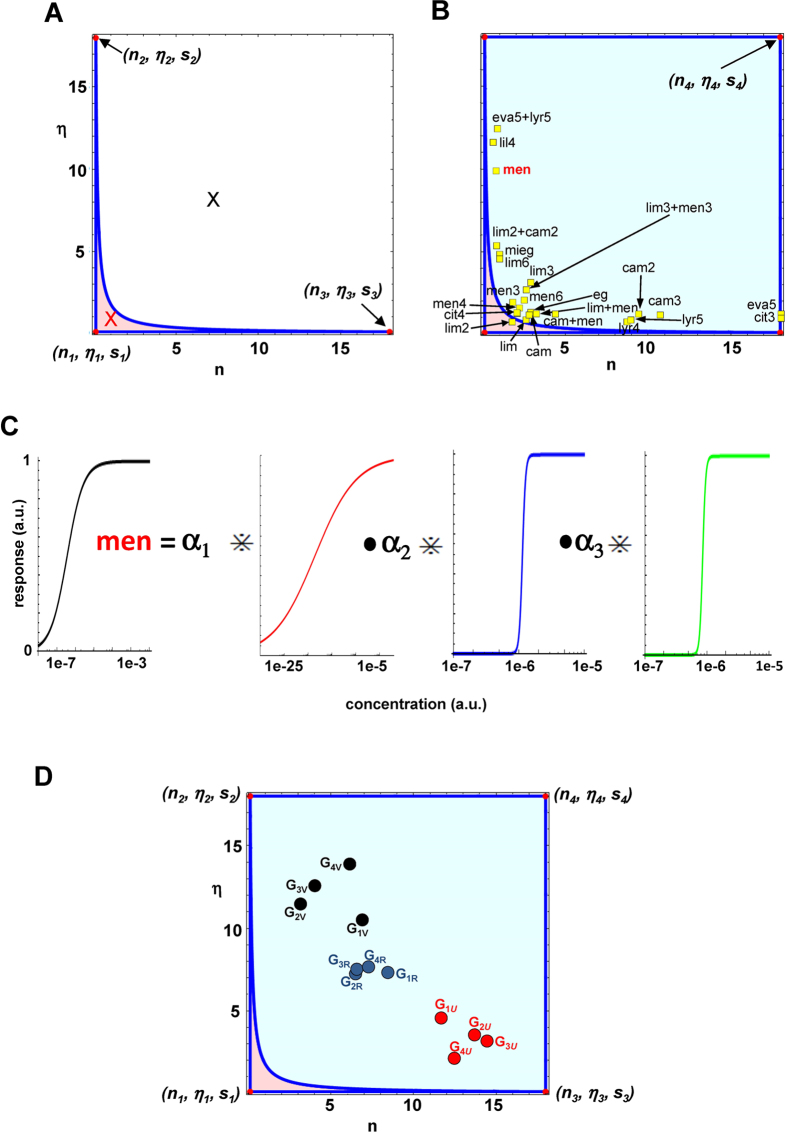
The odor response space can be generated by a three vector basis. (**A**) graphical visualization of one of the basis defined by Eq. (28) (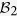 in the text, red dots) and the odor response space it can generate (pink area indicated by the convex hull); odor responses outside the pink area cannot be obtained from this basis. (**B**) using a second basis (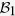 in the text, red dots) allows to cover the complementary odor response space left out by 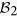; Symbols represent the {*n, η*} parameters best fitting the experimental response of OSNs to different odors (experimental data taken from ref. 5 for *cam, lim, eva, lyr* and *men*, ref. 6 for *lil*, and *cit*, ref. 7 for mieg and *eg*; red colored response to men was chosen as a typical example for panel C; (**C**) response of an OSN to menthol (*men*) obtained as a combination of one of the basis of primary odor responses (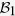), using the same *s* = 10^6^; the values of the coefficients calculated from Eq. (22) are: *α*_1_ = 0.14, *α*_2_ = 0.015, *α*_3_ = 0.18; (**D**) Black and Red symbols represent two odors; in this case we assumed that each odor activated the same four types of OSN in different ways (*G*_1*U*_ …*G*_4*U*_; *G*_1*V*_ …*G*_4*V*_); Blue symbols represent the response to their mixture; see Appendix A.6 for a complete list of parameter values.

The major result of our analysis is thus the expression of an odor response as a combination of three primary responses


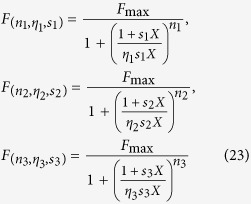


with positive scalar coefficients fulfilling Eqs. (20)–(22), 35, 29. However, because of the constraints defined by Eq. (22), no triple can cover the whole space. In order to have a better visualization of the odor response space, we represent it in the square defined by (positive) values of *n* and *η*, as in Fig. 6. This is allowed because two responses with the same *n* and *η* but different *s* (i.e. concentration) behave as the same odor at different concentrations. The condition is similar, to some extent, to color brightness or sound level; a change in an odor concentration does not change its quality. Combinations in the odor response space thus correspond to convex combinations through the map


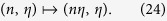


To see the consequences of this result, consider the basis formed by vectors with *n* and *η* values covering the range of the experimental data we considered in this work^5^,^6^,^7^


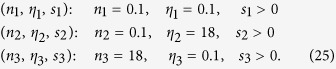


The set of odor responses that can be expressed as a combination of them is represented by those with parameters inside the area defined by the hull (pink area in Fig. 6A) formed by the three vectors above (red circles in Fig. 6A), shaped by Eq. (24). In general, the whole odor response space can be considered as constrained by upper and lower bounds for *n, η*, i.e.


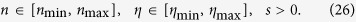


A simple look at Fig. 6A reveals that a basis formed by a triple cannot generate all possible responses, since all those mapped by (*n, η*) values outside the hull (e.g. that represented by the black symbol in Fig. 6A) cannot be obtained. However, it is pretty clear that the hull defined by the four vectors


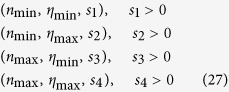


covers the whole experimental space, as schematically represented in Fig. 2B. It can be analytically demonstrated (see Appendix A.5) that these four vectors allow to build the following two bases


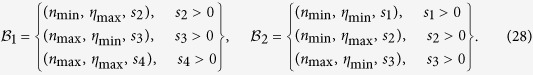


In Fig. 6B we plot several {*n, η*} pairs obtained by fitting experimental odor responses, and a typical example of a response obtained as a combination of three primary responses (the 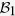 set) is shown in Fig. 6C for menthol (men). Taking into account the response to an odor by a set of OSN types may require either one of the basis defined by Eq. (28).

To illustrate the possible practical consequences of this approach, consider the problem of synthesizing a target odor, *O*_*R*_, smelling let’s say as a rose. This would be represented by the set of OSN responses that can be considered essential to smell a rose (*G*_1*R*_ …*G*_4*R*_, blue symbols in Fig. 6D). Our analysis predicts that it could be conveniently obtained with a mixture of odors activating the same OSNs (red and black symbols in Fig. 3D) combined with coefficients that can be mathematically calculated (see Appendix A.6).

## Conclusions

The main point of our work is thus that any OSN response can be obtained as a combination of primary responses defined by Eq. (27). This result is general and applies no matter how many odor components are present in the odorant, provided that their response curve can be represented as Hill functions. It should be stressed that this is an empirical heuristic model, since it has not been possible (so far) to derive our equations from a specific biophysical/biochemical kinetic scheme. This would be the natural continuation of this work. However, from a physiological point of view, these results suggest that the receptors expressed in olfactory sensory neurons have evolved in such a way to differentiate their relative affinity to three primary types of possible responses that cover the entire odor response space. Given the nature of the problem studied here (OSNs peak activity as a function of odor concentration) the temporal features of the response during a sniff cycle^9^ cannot be taken into account at this stage of the model. In principle, it would be possible to define the response as a function of time (and concentration) and derive the temporal dynamics of the mixture, but this was somewhat out of the scope of this paper. From our analysis, it finally follows that odorants could be classified using the objective properties of the dose-response curves of the OSNs they activate, rather than with the more subjective and less precise perceptual attributes. This approach also opens the possibility for an objective way to manipulate/mix odorants to obtain specific properties from their mixtures.

## Additional Information

**How to cite this article**: Marasco, A. *et al*. Predicting the response of olfactory sensory neurons to odor mixtures from single odor response. *Sci. Rep.* **6**, 24091; doi: 10.1038/srep24091 (2016).

## Supplementary Material

Supplementary Information
