# Supplementary material for: Predicting the response of olfactory sensory neurons to odor mixtures from single odor response
Source: Sci Rep. 2016 Apr 7;6:24091. doi: 10.1038/srep24091 (PMC4823664; doi:10.1038/srep24091)
Supplement: Supplementary Information [file srep24091-s1.pdf]

# Predicting the response of olfactory sensory neurons to odor mixtures from single odor response

A. Marasco, A. De Paris, A. Migliore

## Appendix A.1

In [1]-[2] and [3] two different models were proposed, to take into account the responses $F_U$ and $F_{U+V}$ to concentrations of an odorant $U$ and of a binary mixture $U+V$, respectively. In this appendix we discuss some features of those models, in view of the empirical properties mentioned in the main text. To turn those experimental qualitative descriptions into rigorous mathematical definitions, one should take into account that they involve terms that are not necessarily consensual in the literature, or that cannot be easily expressed into mathematical relations. For this reason we will focus the analysis on the three basic behaviours of *suppression*, *inhibition* and *synergy* (see [5],[2]).

In most cases, the responses $F_U$, $F_V$ to single odorants, and $F_{U+V}$ to the mixture, are compared *at the same concentration*. This means that if a mixture is performed at a certain ratio $r > 0$, then is $U = rV$, and the response $F_{U+V}(U, V) = F_{U+V}(rV, V)$ is relative to the concentration $C := U + V = (1 + r)V$. Henceforth, it is compared with the responses $F_U(C)$ and $F_V(C)$. In this context, we say that *inhibition* happens when

$$F_{U+V}(U, V) < \min\{F_U(C), F_V(C)\} ,$$

*synergy* when

$$F_{U+V}(U, V) > \max\{F_U(C), F_V(C)\},$$

and *suppression* when $F_{U+V}(U, V)$ is (strictly) intermediate between $F_U(C)$, $F_V(C)$. Hence we ignore the comparison of $F_{U+V}(U, V)$ with the sum $F_U(U) + F_V(V)$.

The above interpretation will mainly be used in Appendix A3. In the main text we explain that a slightly different interpretation is also worthy of consideration. It basically consists in considering $V = C/(1 + r)$ in place of $C$, in the response to a mixture. These interpretations do not conflict in the asymptotic region.

- **Rospars et al. models**

In [1] and [2], the authors proposed the following models

$$F_U = \frac{F_{MU}}{1+\left(\frac{K_U}{U}\right)^n}, \quad F_{U+V}(U,V) = \frac{F_{MU}\left(\dfrac{U}{K_U}\right)^n + F_{MV}\left(\dfrac{V}{K_V}\right)^n}{1+\left(\dfrac{U}{K_U}\right)^n + \left(\dfrac{V}{K_V}\right)^n} \tag{1}$$

where $n$ is a Hill coefficient, $K_U, K_V, F_{MU}$ and $F_{MV}$ are constants (see [2]).

- **Cruz and Lowe models**
  In [3] the authors, instead of (1), proposed the following models

$$F_U = \frac{F_{\max}}{1+\dfrac{1}{\eta_U^n}\left(1+\dfrac{K_U}{U}\right)^n}, \quad F_{U+V}(U,V) = \frac{F_{\max}}{1+\left[\dfrac{1+\dfrac{U}{K_U}+\dfrac{V}{K_V}}{\eta_U\dfrac{U}{K_U}+\eta_V\dfrac{V}{K_V}}\right]^n}, \tag{2}$$

  where $n$ is a Hill coefficient, $F_{\max}$ is the maximal response, $\eta_U$ and $\eta_V$ are the efficiencies of activation of transduction by each odorant.

Equations $(1)_2$ and $(2)_2$ are both functions of two compounds in concentration $U$ and $V$. However, following the experimental protocol, it is useful to analyze them under the condition

$$U = rV, \tag{3}$$

where $r$ is a positive constant.

**Rospars et al. binary mixture model**

In [2] is proved that Eq. $(1)_2$ becomes

$$F_{U+V}(rV,V) = \frac{F_{Mp}}{1+\left(\frac{K_p}{(1+r)V}\right)^n}, \quad F_{U+V}(M) = \frac{F_{Mp}}{1+\left(\frac{K_p}{M}\right)^n}, \tag{4}$$

where $M = U + V$ and

$$F_{Mp} = \frac{F_{MU}r^nK_V^n + F_{MV}K_U^n}{(K_U^n + r^nK_V^n)}, \quad K_p = (1+r)\,K_UK_V\sqrt[n]{\frac{1}{r^nK_V^n+K_U^n}}. \tag{5}$$

Then, $F_{Mp}$ is the asymptotic value of function $F_{U+V}(rV,V)$, the concentration of $V$ (resp. of $M$) at half maximum response $F_{Mp}/2$ is $K_p/(1+r)$ (resp. $K_p$). Moreover, the asymptotic value of function $F_{U+V}$ is always located between the single-odorant curves. In fact, from (5) *for all* $r$ we obtain

$$\min(F_{MU}, F_{MV}) \le F_{Mp} \le \max(F_{MU}, F_{MV}). \tag{6}$$

**Cruz and Lowe binary mixture model**

Analogously, if the concentrations $U$ and $V$ of the odorants are such that $U = rV$ Eq. $(2)_2$ becomes

$$F_{U+V}(U,V) = F_{U+V}(rV,V) = \frac{F_{\max}}{1 + \dfrac{1}{\bar{\eta}^n}\left(1 + \dfrac{\bar{K}}{V}\right)^n} \tag{7}$$

where the parameters $\bar{\eta} = \bar{\eta}(r, \eta_U, \eta_V, K_U, K_V), \bar{K} = \bar{K}(r, K_U, K_V)$ assume the form

$$\bar{\eta} = \frac{\eta_U r K_V + \eta_V K_U}{K_U + r K_V}, \quad \bar{K} = \frac{K_U K_V}{K_U + r K_V}. \tag{8}$$

In this case, the asymptotic value of function $F_{U+V}(rV,V)$ is

$$\frac{F_{\max}}{1 + \dfrac{1}{\bar{\eta}^n}} = \frac{\bar{\eta}^n F_{\max}}{1 + \bar{\eta}^n}, \tag{9}$$

and the concentration of $V$ at half maximun response $\dfrac{\bar{\eta}^n F_{\max}}{2(1 + \bar{\eta}^n)}$ is

$$\frac{\bar{K}}{\sqrt[n]{2 + \bar{\eta}^n} - 1} \tag{10}$$

From (8) we obtain

$$\min(\eta_U, \eta_V) \leq \bar{\eta} \leq \max(\eta_U, \eta_V)$$

and owing to (9) *for all* $r$ we have

$$\min\left(\frac{F_{\max}}{1 + \dfrac{1}{\eta_U^n}}, \frac{F_{\max}}{1 + \dfrac{1}{\eta_V^n}}\right) \leq \frac{F_{\max}}{1 + \dfrac{1}{\bar{\eta}^n}} \leq \max\left(\frac{F_{\max}}{1 + \dfrac{1}{\eta_U^n}}, \frac{F_{\max}}{1 + \dfrac{1}{\eta_V^n}}\right). \tag{11}$$

Then, owing to (6) and (11), both the models (4) and (7) cannot reproduce mixtures exhibiting synergy or inhibition in their maximal response.

# Appendix A.2

## Model for binary mixture

In order to find a model able to describe each type of interaction among two single odorant it is necessary to expand the space of allowed Hill functions. To this purpose, we assume that the Hill coefficient in the mixture model $(2)_2$ can depend on the odorants. Note that there are experimental findings showing that the response of the same OSN to different odorants may exhibit different Hill coefficients (see [7]). Then, starting from Eq. $(2)_1$ for the odorants $U$ and $V$

$$F_U(U) = \frac{F_{\max}}{1 + \frac{1}{\eta_U^{n_U}}\left(1 + \frac{K_U}{U}\right)^{n_U}}, \quad F_V(V) = \frac{F_{\max}}{1 + \frac{1}{\eta_V^{n_V}}\left(1 + \frac{K_V}{V}\right)^{n_V}}, \tag{12}$$

we propose the following **model for binary mixture**

$$F_{U+V}(U,V) = \frac{F_{\max}}{1 + \left[\dfrac{1 + \dfrac{U}{K_U} + \dfrac{V}{K_V}}{\eta_U \dfrac{U}{K_U} + \eta_V \dfrac{V}{K_V}}\right]^{n_{U+V}}} \tag{13}$$

where

$$n_{U+V} = \frac{n_U \eta_U K_V U + n_V \eta_V K_U V}{\eta_U K_V U + \eta_V K_U V}. \tag{14}$$

Equation (13) is a function of two compounds in concentration $U$ and $V$. However, if $U = rV$ where $r$ is a constant, we obtain

$$\frac{F_{\max}}{1 + \frac{1}{\bar{\eta}^{\bar{n}}}\left(1 + \frac{\bar{K}}{V}\right)^{\bar{n}}} \tag{15}$$

where the parameters $\bar{\eta} = \bar{\eta}\left(r, \eta_U, \eta_V, K_U, K_V\right), \bar{K} = \bar{K}\left(r, K_U, K_V\right)$ and $\bar{n} = \bar{n}\left(r, n_U, n_V, \eta_U, \eta_V, K_U, K_V\right)$ assume the form

$$\bar{\eta} = \frac{\eta_U r K_V + \eta_V K_U}{K_U + r K_V}, \quad \bar{K} = \frac{K_U K_V}{K_U + r K_V}, \quad \bar{n} = \frac{r n_U \eta_U K_V + n_V \eta_V K_U}{r \eta_U K_V + \eta_V K_U}. \tag{16}$$

We note that if $n_U = n_V$, then (13) and (15) become $(2)_2$ and (7), respectively.

The asymptotic value of function (15) is

$$\frac{F_{\max}}{1 + \frac{1}{\bar{\eta}^{\bar{n}}}} = \frac{\bar{\eta}^{\bar{n}} F_{\max}}{1 + \bar{\eta}^{\bar{n}}}, \tag{17}$$

and the concentration of $V$ at half maximun response $\dfrac{\bar{\eta}^{\bar{n}} F_{\max}}{2\left(1 + \bar{\eta}^{\bar{n}}\right)}$ is

$$\frac{\bar{K}}{\sqrt[\bar{n}]{2 + \bar{\eta}^{\bar{n}}} - 1} \tag{18}$$

## Model for mixture of N odorants

Equation (13) can be easily generalized to a **model for mixture of N odorants**

$$F_{MIX}\left(U_1,...,U_N\right)=\frac{F_{\max}}{1+\left[\dfrac{1+\displaystyle\sum_{i=1}^{N}\frac{U_i}{K_{U_i}}}{\displaystyle\sum_{i=1}^{N}\eta_{U_i}\frac{U_i}{K_{U_i}}}\right]^{n_{MIX}}} \tag{19}$$

where

$$n_{MIX}=\frac{\displaystyle\sum_{i=1}^{N}n_{U_i}\eta_{U_i}\frac{U_i}{K_{U_i}}}{\displaystyle\sum_{i=1}^{N}\eta_{U_i}\frac{U_i}{K_{U_i}}}. \tag{20}$$

Similarly, setting for all $i\in\{1,...,N-1\}\,,U_i=r_iU_N$ where $r_i$ are positive constants, we obtain

$$F_{MIX}\left(r_1U_N,...,U_N\right)=\frac{F_{\max}}{1+\dfrac{1}{\eta_{MIX}^{n_{MIX}}}\left(1+\dfrac{K_{MIX}}{U_N}\right)^{n_{MIX}}} \tag{21}$$

where

$$\begin{aligned}\eta_{MIX}&=\frac{\displaystyle\sum_{i=1}^{N-1}\frac{r_i}{K_{U_i}}\eta_{U_i}+\frac{1}{K_{U_N}}\eta_{U_N}}{\displaystyle\sum_{i=1}^{N-1}\frac{r_i}{K_{U_i}}+\frac{1}{K_{U_N}}},\quad K_{MIX}=1/\left(\sum_{i=1}^{N-1}\frac{r_i}{K_{U_i}}+\frac{1}{K_{U_N}}\right),\\ n_{MIX}&=\frac{\displaystyle\sum_{i=1}^{N-1}n_{U_i}\eta_{U_i}\frac{r_i}{K_{U_i}}+n_{U_N}\eta_{U_N}\frac{1}{K_{U_N}}}{\displaystyle\sum_{i=1}^{N-1}\eta_{U_i}\frac{r_i}{K_{U_i}}+\eta_{U_N}\frac{1}{K_{U_N}}}\end{aligned} \tag{22}$$

# Appendix A.3: Analysis of the proposed mixture model

For applicative purposes, one might be interested in knowing a priori for which mixtures our model (13), (14) predicts inhibition, synergy or suppression, as described at the beginning of Appendix A1. Here we give details for the whole range of concentrations; namely, we look for the three ranges of values of $U, K_U, \eta_U, n_U, V, K_V, \eta_V, n_V$, for which those qualitative behaviours occur. This problem obviously consists in solving (rather cumbersome) inequalities that arise by comparing the values $F_{U+V}(U,V)$, $F_U(C)$, $F_V(C)$ given by (13) and (12) with $C := U + V$.

To use

$$r := U/V, \qquad C = U + V$$

instead of the pair $U, V$ is perhaps more meaningful and mathematically simpler. Because of the inverse formulas

$$U = \frac{rC}{1+r}, \qquad V = \frac{C}{1+r}$$

we have no loss, nor substantial bias of information.

Note also that if one gets $n_U, n_V$ multiplied by the same (positive) constant, the behaviour stays the same. Hence the ranges can be described using the parameter

$$\rho := n_U/n_V$$

instead of the pair $(n_U, n_V)$. When $n_U = n_V$ we have the Cruz and Lowe model, which has already been discussed. Hence we assume $n_U \neq n_V$. Moreover, to avoid an unnecessary long description, we assume that $U$ denotes the odorant with the least Hill coefficient (exponent $n$):

$$\boxed{n_U < n_V},$$

that is, $\rho < 1$. Hence, to test parameters against the list of cases we are going to display, one should keep in mind that if $n_U > n_V$, then $U$ and $V$ have to be exchanged (either in the data or in the list).

It will also be convenient to set

$$b_U := \frac{K_U + C}{\eta_U C}\ , \qquad b_V := \frac{K_V + C}{\eta_V C}$$

and to consider the function $y = x + x \ln x$. This function has an absolute minimum $(x, y) = (\exp(-2), -\exp(-2))$, and it is increasing for $x \geq \exp(-2)$. We shall denote by $\varphi(y)$ the (partial) inverse defined for $y \geq -\exp(-2)$ (with values $x \geq \exp(-2)$). To better understand the cases in the list below, one should take into account that $y > \varphi(y) > 1$ when $y > 1$ and $y < \varphi(y) < 1$ when $y < 1$.

For the sake of brevity, we shall disregard some edge cases: in the list below we shall always use strict inequalities and we also assume

$$\eta_V K_U K_V (K_U + C) \neq \eta_U K_U K_V (K_V + C)$$

(that is, $F_{U+V}(rC/(1+r), C/(1+r))$ is not constant with respect to $r$). We also renounce to discuss some of the cases that are more cumbersome to be analysed.

- When $1 < b_U < b_V$ we have **suppression** for all $\rho, r$ (under the standing assumptions $0 < \rho < 1$, $r > 0$).
- When $b_U > 1$ and $(1 <)\varphi(b_U) < b_V < b_U$:
  - when
    $$(1 >)\rho > \frac{\eta_V (K_U + C) - \eta_U \eta_V C (b_U + b_U \ln b_U)}{\eta_U (K_V + C) - \eta_U \eta_V C (b_U + b_U \ln b_U)} (> 0)$$
    we have **suppression** for all $r (> 0)$;
  - when
    $$(0 <)\frac{\ln b_V}{\ln b_U} < \rho < \frac{\eta_V (K_U + C) - \eta_U \eta_V C (b_U + b_U \ln b_U)}{\eta_U (K_V + C) - \eta_U \eta_V C (b_U + b_U \ln b_U)} (< 1)$$
    there exists exactly one value $r_U > 0$ for which
    $$F_{U+V}(r_U C/(1+r_U), C/(1+r_U)) = F_U(C)$$
    (to be determined numerically) and we have
    * **suppression** for $(0 <)r < r_U$,
    * **inhibition** for $r > r_U$;
  - when
    $$(0 <)\frac{\eta_V (K_U + C) - \eta_U \eta_V C (b_V + b_V \ln b_V)}{\eta_U (K_V + C) - \eta_U \eta_V C (b_V + b_V \ln b_V)} < \rho < \frac{\ln b_V}{\ln b_U} (< 1)$$
    there exists exactly one value $r_V > 0$ for which
    $$F_{U+V}(r_V C/(1+r_V), C/(1+r_V)) = F_V(C)$$
    (to be determined numerically) and we have
    * **inhibition** for $(0 <)r < r_V$,
    * **suppression** for $r > r_V$;
  - when
    $$(0 <)\rho < \frac{\eta_V (K_U + C) - \eta_U \eta_V C (b_V + b_V \ln b_V)}{\eta_U (K_V + C) - \eta_U \eta_V C (b_V + b_V \ln b_V)}$$
    we have **suppression** for all $r (> 0)$.

- When $b_U > 1$ and $1 < b_V < \varphi(b_U)$:
  - when
  $$(1 >)\rho > \frac{\eta_V (K_U + C) - \eta_U \eta_V C (b_U + b_U \ln b_U)}{\eta_U (K_V + C) - \eta_U \eta_V C (b_U + b_U \ln b_U)} (> 0)$$
  we have **suppression** for all $r (> 0)$;
  - when
  $$(0 <) \frac{\ln b_V}{\ln b_U} < \rho < \frac{\eta_V (K_U + C) - \eta_U \eta_V C (b_U + b_U \ln b_U)}{\eta_U (K_V + C) - \eta_U \eta_V C (b_U + b_U \ln b_U)} (< 1)$$
  there exists exactly one value $r_U > 0$ for which
  $$F_{U+V} (r_U C/(1 + r_U), C/(1 + r_U)) = F_U(C)$$
  (to be determined numerically) and we have
    * **suppression** for $(0 <) r < r_U$,
    * **inhibition** for $r > r_U$;
  - when
  $$(0 <)\rho < \frac{\ln b_V}{\ln b_U} (< 1)$$
  there exists exactly one value $r_V > 0$ for which
  $$F_{U+V} (r_V C/(1 + r_V), C/(1 + r_V)) = F_V(C)$$
  (to be determined numerically) and we have
    * **inhibition** for $(0 <) r < r_V$,
    * **suppression** for $r > r_V$.
- When $b_U > 1$ and $(0 <) b_V < 1$:
  - when
  $$(1 >)\rho > \frac{\eta_V (K_U + C) - \eta_U \eta_V C (b_U + b_U \ln b_U)}{\eta_U (K_V + C) - \eta_U \eta_V C (b_U + b_U \ln b_U)} (> 0)$$
  we have **suppression** for all $r (> 0)$;
  - when
  $$(0 <)\rho < \frac{\eta_V (K_U + C) - \eta_U \eta_V C (b_U + b_U \ln b_U)}{\eta_U (K_V + C) - \eta_U \eta_V C (b_U + b_U \ln b_U)} (< 1)$$
  there exists exactly one value $r_U > 0$ for which
  $$F_{U+V} (r_U C/(1 + r_U), C/(1 + r_U)) = F_U(C)$$
  (to be determined numerically) and we have

    * **suppression** for $(0<)r<r_U$,
    * **inhibition** for $r>r_U$.

- When $(0<)b_U<1$ and $b_V>1$:
  - when

$$(1>)\rho>\frac{\eta_V\left(K_U+C\right)-\eta_U\eta_V C\left(b_U+b_U\ln b_U\right)}{\eta_U\left(K_V+C\right)-\eta_U\eta_V C\left(b_U+b_U\ln b_U\right)}(>0)$$

    we have **suppression** for all $r(>0)$;
  - when

$$(0<)\rho<\frac{\eta_V\left(K_U+C\right)-\eta_U\eta_V C\left(b_U+b_U\ln b_U\right)}{\eta_U\left(K_V+C\right)-\eta_U\eta_V C\left(b_U+b_U\ln b_U\right)}(<1)$$

    there exists exactly one value $r_U>0$ for which

$$F_{U+V}\left(r_U C/(1+r_U),C/(1+r_U)\right)=F_U(C)$$

    (to be determined numerically) and we have
    * **suppression** for $(0<)r<r_U$,
    * **synergy** for $r>r_U$.

- When $b_U<1$ and $\varphi(b_U)<b_V<1$:
  - when

$$(1>)\rho>\frac{\eta_V\left(K_U+C\right)-\eta_U\eta_V C\left(b_U+b_U\ln b_U\right)}{\eta_U\left(K_V+C\right)-\eta_U\eta_V C\left(b_U+b_U\ln b_U\right)}(>0)$$

    we have **suppression** for all $r(>0)$;
  - when

$$(0<)\frac{\ln b_V}{\ln b_U}<\rho<\frac{\eta_V\left(K_U+C\right)-\eta_U\eta_V C\left(b_U+b_U\ln b_U\right)}{\eta_U\left(K_V+C\right)-\eta_U\eta_V C\left(b_U+b_U\ln b_U\right)}(<1)$$

    there exists exactly one value $r_U>0$ for which

$$F_{U+V}\left(r_U C/(1+r_U),C/(1+r_U)\right)=F_U(C)$$

    (to be determined numerically) and we have
    * **suppression** for $0<r<r_U$,
    * **synergy** for $r>r_U$;
  - when $(0<)\rho<\ln b_V/\ln b_U(<1)$ there exists exactly one value $r_V>0$ for which

$$F_{U+V}\left(r_V C/(1+r_V),C/(1+r_V)\right)=F_V(C)$$

    (to be determined numerically) and we have

* **synergy** for $0 < r < r_V$,
* **suppression** for $r > r_V$.

- When $\exp(-2) < b_U < 1$ and $b_U < b_V < \varphi(b_U)$:
  - when
    $$(1 >)\rho > \frac{\eta_V (K_U + C) - \eta_U \eta_V C (b_U + b_U \ln b_U)}{\eta_U (K_V + C) - \eta_U \eta_V C (b_U + b_U \ln b_U)}(> 0)$$
    we have **suppression** for all $r(> 0)$;
  - when
    $$(0 <)\frac{\ln b_V}{\ln b_U} < \rho < \frac{\eta_V (K_U + C) - \eta_U \eta_V C (b_U + b_U \ln b_U)}{\eta_U (K_V + C) - \eta_U \eta_V C (b_U + b_U \ln b_U)}(< 1)$$
    there exists exactly one value $r_U > 0$ for which
    $$F_{U+V}(r_U C/(1 + r_U), C/(1 + r_U)) = F_U(C)$$
    (to be determined numerically) and we have
    * **suppression** for $(0 <)r < r_U$,
    * **synergy** for $r > r_U$;
  - when
    $$(0 <)\frac{\eta_V (K_U + C) - \eta_U \eta_V C (b_V + b_V \ln b_V)}{\eta_U (K_V + C) - \eta_U \eta_V C (b_V + b_V \ln b_V)} < \rho < \frac{\ln b_V}{\ln b_U}(< 1)$$
    there exists exactly one value $r_V > 0$ for which
    $$F_{U+V}(r_V C/(1 + r_V), C/(1 + r_V)) = F_V(C)$$
    (to be determined numerically) and we have
    * **synergy** for $(0 <)r < r_V$,
    * **suppression** for $r > r_V$;
  - when
    $$(0 <)\rho < \frac{\eta_V (K_U + C) - \eta_U \eta_V C (b_V + b_V \ln b_V)}{\eta_U (K_V + C) - \eta_U \eta_V C (b_V + b_V \ln b_V)}(< 1)$$
    we have **suppression** for all $r(> 0)$.
- When $(0 <)b_U < \exp(-2)$ and $\exp(-2) < b_V < \varphi(b_U)$ the analysis is more involved and we omit the result.
- When $(0 <)b_U < b_V < \exp(-2)$:

– when

$$(1 >)\rho > \frac{\eta_V\left(K_U + C\right) - \eta_U \eta_V C\left(b_V + b_V \ln b_V\right)}{\eta_U\left(K_V + C\right) - \eta_U \eta_V C\left(b_V + b_V \ln b_V\right)}(> 0)$$

we have **suppression** for all $r(> 0)$;

– when

$$(0 <)\frac{\ln b_V}{\ln b_U} < \rho < \frac{\eta_V\left(K_U + C\right) - \eta_U \eta_V C\left(b_V + b_V \ln b_V\right)}{\eta_U\left(K_V + C\right) - \eta_U \eta_V C\left(b_V + b_V \ln b_V\right)}(< 1)$$

there exists exactly one value $r_V > 0$ for which

$$F_{U+V}\left(r_V C/(1 + r_V), C/(1 + r_V)\right) = F_V(C)$$

(to be determined numerically) and we have

  * **inhibition** for $(0 <)r < r_V$,
  * **suppression** for $r > r_V$;

– when

$$(0 <)\frac{\eta_V\left(K_U + C\right) - \eta_U \eta_V C\left(b_U + b_U \ln b_U\right)}{\eta_U\left(K_V + C\right) - \eta_U \eta_V C\left(b_U + b_U \ln b_U\right)} < \rho < \frac{\ln b_V}{\ln b_U}(< 1)$$

there exists exactly one value $r_U > 0$ for which

$$F_{U+V}\left(r_U C/(1 + r_U), C/(1 + r_U)\right) = F_U(C)$$

(to be determined numerically) and we have

  * **suppression** for $0 < r < r_U$,
  * **inhibition** for $r > r_U$;

– when

$$(0 <)\rho < \frac{\eta_V\left(K_U + C\right) - \eta_U \eta_V C\left(b_U + b_U \ln b_U\right)}{\eta_U\left(K_V + C\right) - \eta_U \eta_V C\left(b_U + b_U \ln b_U\right)}(< 1)$$

we have **suppression** for all $r(> 0)$.

- When $(0 <)b_V < b_U < 1$ we have **suppression** for all $(0 <)\rho(< 1)$, $r(> 0)$.

An important consequence of the above analysis is that the model cannot exhibit synergy for concentrations lower than the concentration that gives the half value response for $U$ (i.e. the odorant with lower Hill coefficient). Indeed, note that none of the cases for which $b_U > 1$ gives synergy. Hence, if for a concentration $C$ we have synergy, it must be $b_U \leq 1$, therefore ${b_U}^{n_U} \leq 1$, which leads to

$$F_U(C) = \frac{F_{\max}}{1 + {b_U}^{n_U}} \geq \frac{F_{\max}}{2}.$$

Since $F_{\max}$ clearly exceeds the maximum response for $U$, and $F_U$ is an increasing function, we deduce that $C$ must exceed the concentration that gives the half value response for $U$.

# Appendix A.4: Equations for specific experimental protocols

## Experiments in Rospars et al., 2008

In [2], for each OSN the odorants $U, V$, and $U+V$ were tested at the concentrations

$$U=\frac{M_0^U}{2d},\quad V=\frac{M_0^V}{2d},\quad M=U+V=\frac{M_0^V}{2d}+\frac{M_0^U}{2d},$$

where $d\in\left\{10^{i/4}, i=0,...,11\right\}$ is the dilution, and $M_0^U$ e $M_0^V$ are the molarities of saturing vapors (see Table 1 in [2]). In this way, in each experiment results

$$U=rV,\quad M=U+V=(1+r)V, \tag{23}$$

where the ratio

$$r=\frac{M_0^U}{M_0^V}$$

remains constant at different dilutions.

The experimental raw data of dose-response curves for the odorants $U, V$, and the binary mixture $U+V$ are given in the form (JP Rospars personal communication)

$$\left(\frac{M_0^U}{2d}, F_U\right),\quad \left(\frac{M_0^V}{2d}, F_V\right),\quad \left(\frac{M_0^U}{2d}+\frac{M_0^V}{2d}, F_{U+V}\right). \tag{24}$$

By fitting the experimental data (24) for the single odorants $U$ and $V$ we determine the dose-response functiones $F_U$ and $F_V$ (see Eq.(12)), whereas the response $F_{U+V}$ is determined as a function of the mixture concentration $M=U+V$ by mean of Eq.(13) in which $V=M/(1+r)$, i.e.

$$F_{U+V}\left(M\right)=\frac{1}{1+\frac{1}{\bar{\eta}^{\bar{n}}}\left(1+\frac{\bar{K}}{M/(1+r)}\right)^{\bar{n}}} \tag{25}$$

where the parameters $\bar{\eta}, \bar{K}$ and $\bar{n}$ assume the form

$$\bar{\eta}=\frac{\eta_U rK_V+\eta_V K_U}{K_U+rK_V},\quad \bar{K}=\frac{K_U K_V}{K_U+rK_V},\quad \bar{n}=\frac{rn_U\eta_U K_V+n_V\eta_V K_U}{r\eta_U K_V+\eta_V K_U}. \tag{26}$$

## Experiments in Cruz e Lowe, 2013

Differently to [2], in [3] the mixture is tested at a fixed concentration of one of the components. In this case, after determining by fitting the numerical values of the parameters $n_U, \eta_U, K_U, n_V, \eta_V, K_V$, using Eq.(13) we obtain the response $F_{U+V}$ to concentration of mixture $U+V$ for a fixed value of the odorant $V$. In

detail, by fixing the concentration $C$ for the odorant $V$ we obtain the response $F_{U+V}$ as a function of concentration of odorant $U$

$$F_{U+V}\left(U,C\right)=\frac{1}{1+\left[\frac{1+\frac{U}{K_U}+\frac{C}{K_V}}{\eta_U\frac{U}{K_U}+\eta_V\frac{C}{K_V}}\right]^{n_{U+V}}} \tag{27}$$

where

$$n_{U+V}=\frac{n_U\eta_U K_V U+n_V\eta_V K_U C}{\eta_U K_V U+\eta_V K_U C}.$$

For example, in the cases of the mixture $\mathbf{EG}+\mathbf{MIEG(1.05)}$ (see Fig.4, bottom right panel) the dose-response function is

$$F_{U+V}\left(X,1.05\right)=\frac{1}{1+\left[\frac{1+\frac{X}{K_U}+\frac{1.05}{K_V}}{\eta_U\frac{X}{K_U}+\eta_V\frac{1.05}{K_V}}\right]^{\frac{n_U\eta_U K_V X+1.05n_V\eta_V K_U}{\eta_U K_V X+1.05\eta_V K_U}}}$$

where $X$ is the concentration of odorant **EG**.

## Performance of the proposed mixture model

In order to evaluate the performance of the proposed mixture model, we use the *mean square error (MSE)* and the *mean absolute percentage error (MAPE)*[1] to compare the experimental data with the predicted data. The MAPE classification leves are shown in Supp. Table S1 [6]

| MAPE% | **Error classification** |
|---|---|
| $< 10$ | Highly accurate |
| $10-20$ | Good |
| $20-50$ | Reasonable |
| $> 50$ | Inaccurate |

**Supp. Table S1:** Classification levels of MAPE

[1] We recall that

$$MAPE=\frac{1}{n}\sum_{i=1}^{n}\left|\frac{E_i-P_i}{P_i}\right|100\%$$

where $E_i$ and $P_i$ are respectively the experimental and predicted values.

MSE and MAPE for all set of experimental data in Figs.4-5

| *Fig. 4* | **lim** | **men** | **lim + men** |
|---|---|---|---|
| *MSE* | $5.825 \times 10^{-4}$ | $2.118 \times 10^{-3}$ | $4.583 \times 10^{-3}$ |
| *MAPE* | 10.273 | 9.972 | 18.484 |

| | **cam** | **men** | **cam + men** |
|---|---|---|---|
| *MSE* | $1.953 \times 10^{-4}$ | $2.118 \times 10^{-3}$ | $1.275 \times 10^{-3}$ |
| *MAPE* | 3.632 | 9.972 | 11.687 |

| | **cam** | **lim** | **cam + lim** |
|---|---|---|---|
| *MSE* | $7.005 \times 10^{-4}$ | $9.548 \times 10^{-4}$ | $3.230 \times 10^{-2}$ |
| *MAPE* | 9.115 | 4.857 | 27.570 |

| | **lim** | **lyr** | **lim + lyr** |
|---|---|---|---|
| *MSE* | $7.854 \times 10^{-4}$ | $1.059 \times 10^{-3}$ | $1.155 \times 10^{-2}$ |
| *MAPE* | 10.545 | 29.615 | 39.598 |

| | **eg** | **mieg** | **eg + mieg(1.05)** |
|---|---|---|---|
| *MSE* | $1.638 \times 10^{-3}$ | $3.089 \times 10^{-4}$ | $5.340 \times 10^{-3}$ |
| *MAPE* | 18.398 | 4.079 | 8.431 |

| | **eg** | **mieg** | **eg + mieg(6.45)** |
|---|---|---|---|
| *MSE* | $1.638 \times 10^{-3}$ | $3.089 \times 10^{-4}$ | $1.382 \times 10^{-3}$ |
| *MAPE* | 18.398 | 4.079 | 4.228 |

**Suppl. Table S2:** MSE and MAPE for the experiments in Fig.4

| *Fig. 5* | **lim** | **men** | **lim + men** | **lim + men corrected ($-\mathbf{0.06}\log_{10}\mathbf{M}$)** |
|---|---|---|---|---|
| *MSE* | $1.728 \times 10^{-3}$ | $4.808 \times 10^{-5}$ | $3.840 \times 10^{-2}$ | $3.099 \times 10^{-2}$ |
| *MAPE* | 7.407 | 2.096 | 55.957 | 31.644 |

| | **eva** | **lyr** | **eva + lyr** | **eva + lyr corrected ($\mathbf{0.026}\log_{10}\mathbf{M}$)** |
|---|---|---|---|---|
| *MSE* | $7.403 \times 10^{-3}$ | $7.603 \times 10^{-4}$ | $2.153 \times 10^{-2}$ | $3.645 \times 10^{-2}$ |
| *MAPE* | 10.938 | 13.810 | 33.904 | 19.270 |

| | **cit** | **lil** | **cit + lil** | **cit + lil corrected ($-\mathbf{0.1}\log_{10}\mathbf{M}$)** |
|---|---|---|---|---|
| *MSE* | $3.007 \times 10^{-3}$ | $2.230 \times 10^{-3}$ | $2.449 \times 10^{-2}$ | $2.273 \times 10^{-2}$ |
| *MAPE* | 10.174 | 12.978 | 27.043 | 15.988 |

**Suppl. Table S3:** MSE and MAPE for the experiments in Fig.5

## Appendix A.5

Our purpose in this section is to study from a mathematical point of view the OSN responses modelled by (12) and (13).

Consider first the following function

$$F_{(n,\eta,s)}\left(X\right)=\frac{F_{\max}}{1+\left(\dfrac{1+sX}{\eta sX}\right)^{n}}, \tag{28}$$

where the variable $X$ ranges in the real positive numbers, and the parameters $n, \eta, s$ are positive as well.

Note that when $s = 1/K$ and $X$ is an odorant concentration $(U, V, ...)$ we get (12). In this way OSN responses are labeled by the triples $(n, \eta, s)$,

$$(n,\eta,s)\longleftrightarrow F_{(n,\eta,s)}\left(X\right) \tag{29}$$

and the **odor response space** is

$$\mathcal{ORS}=\left\{\frac{F_{\max}}{1+\left(\dfrac{1+sX}{\eta sX}\right)^{n}}\right\}_{n,\eta,s\in\mathbb{R}^{+}}. \tag{30}$$

Let us introduce two **composition laws**

$$\begin{aligned}&(n,\eta,s)\bullet(n',\eta',s')=\left(\frac{n\eta s+n'\eta's'}{\eta s+\eta's'},\frac{\eta s+\eta's'}{s+s'},s+s'\right),\\&\alpha\circledast(n,\eta,s)=(n,\eta,\alpha s),\quad \alpha\in\mathbb{R}_{+}.\end{aligned} \tag{31}$$

We remark that

- $(n, \eta, s)\bullet(n', \eta', s')$ identifies a mixture of two odorants $(n, \eta, s)$ and $(n', \eta', s')$, i.e.

$$(n,\eta,s)\bullet(n',\eta',s')\longleftrightarrow F_{(n,\eta,s)\bullet(n',\eta',s')}=\frac{F_{\max}}{1+\left(\dfrac{1+(s+s')\,X}{(\eta s+\eta's')\,X}\right)^{\dfrac{n\eta s+n'\eta's'}{\eta s+\eta's'}}}$$

- $\alpha \circledast (n, \eta, s)$, $\alpha \in \mathbb{R}_+$ identifies the odorant $(n, \eta, s)$ at concentration $\alpha X$, i.e.

$$\alpha\circledast(n,\eta,s)\longleftrightarrow\frac{F_{\max}}{1+\left(\frac{1+s(\alpha X)}{\eta s(\alpha X)}\right)^{n}}.$$

The practical meaning of the above operations is rather simple: to mix odorants $(n, \eta, s)$, $(n', \eta', s')$ at a fixed ratio $r$ $(U = rV)$ is equivalent to consider

$$\left[\frac{r}{r+1} \circledast (n, \eta, s)\right] \bullet \left[\frac{1}{r+1} \circledast (n', \eta', s')\right] ,$$

that is, a weighted mean in term of the operations. Some natural properties can easily be checked: for instance, $\bullet$ is commutative and associative. This means, obviously, that a mixture of several odorants does not depend on the way one mixes them, but only on the reciprocal ratios. On the other hand, we can not have 'opposite odorants', because of the positivity constraints. The latter is basically the only obstacle that prevents our operations from defining a vector space structure. To understand why, let us recall that to define a finite-dimesional vector space structure over the real numbers is the same as to define a one-to-one map onto $\mathbb{R}^n$ (actually, an equivalence class of them): the condition to be fulfilled is that the structural operations must correspond, via the map, with the usual addition and scaling of vectors. We shall see below that such a map can be constructed, with the constraint of not being onto $\mathbb{R}^n$ $(n = 3)$.

The space $(\mathcal{ORS}, \bullet, \circledast)$ embeds into the standard topological vector space $\mathbb{R}^3$ by means of the map

$$(n, \eta, s) \mapsto (n\eta s, \eta s, s). \tag{32}$$

Indeed from (32) we obtain that

$$(n, \eta, s) \bullet (n', \eta', s') \mapsto (n\eta s, \eta s, s) + (n'\eta' s', \eta' s', s') \tag{33}$$

$$\alpha \circledast (n, \eta, s) \mapsto \alpha(n\eta s, \eta s, s) \tag{34}$$

The first simple qualitative conclusion suggested from the mathematical point of view is that no privileged odorants arise in the odor space. Indeed, the only 'special' element in $\mathbb{R}^3$ is the zero triple, which is out of the range of the embedding (i.e. $K$ lies at infinity). The special status of the zero triple can be justified by saying that any automorphism (i.e. transformations preserving the mathematical structure) must transform the zero triple into itself. On the contrary, any two nonzero triples can be transformed into each other by some automorphism, and are henceforth equivalent from a structural (mathematical) point of view.

Another interesting feature carried by the vector space structure of $\mathbb{R}^3$ is that the choice of three independent vectors (i.e., a basis) suffices to generate the whole space. The map (32) could be used to carry a similar feature on the odor space,

$$(\alpha_1 \circledast (n_1, \eta_1, s_1)) \bullet (\alpha_2 \circledast (n_2, \eta_2, s_2)) \bullet (\alpha_3 \circledast (n_3, \eta_3, s_3)) \mapsto \sum_{i=1}^{3} \alpha_i \left(n_i \eta_i s_i, \eta_i s_i, s_i\right) , \tag{35}$$

but with some cautions. Indeed, the odor space is mapped only on the positive octant $\mathbb{R}^3_+$, because only positive scalars are allowed in order to have a physically meaningful combinations

$$(\alpha_1 \divideontimes (n_1, \eta_1, s_1)) \bullet (\alpha_2 \divideontimes (n_2, \eta_2, s_2)) \bullet (\alpha_3 \divideontimes (n_3, \eta_3, s_3)) \,. \tag{36}$$

Thus, let us choose three vectors

$$\begin{aligned} \mathbf{e}_1 &= (n_1\eta_1 s_1, \eta_1 s_1, s_1) \,, \\ \mathbf{e}_2 &= (n_2\eta_2 s_2, \eta_2 s_2, s_2) \,, \\ \mathbf{e}_3 &= (n_3\eta_3 s_3, \eta_3 s_3, s_3) \,, \end{aligned} \tag{37}$$

in the positive octant $\mathbb{R}^3_+$, which constitute a basis provided that

$$\Delta \equiv \eta_1\eta_2 \,(n_1 - n_2) - \eta_1\eta_3 \,(n_1 - n_3) + \eta_2\eta_3 \,(n_2 - n_3) \neq 0. \tag{38}$$

Every vector $(n\eta s, \eta s, s) \in \mathbb{R}^3_+$ is uniquely obtained as a linear combination of (37), i.e.

$$\forall (n\eta s, \eta s, s) \quad \exists! \,(\alpha_1, \alpha_2, \alpha_3) : \quad (n\eta s, \eta s, s) = \sum_{i=1}^{3} \alpha_i \,(n_i\eta_i s_i, \eta_i s_i, s_i) \tag{39}$$

The scalars in (39) must be positive and can explicitly be determined as follows:

$$\begin{aligned} \alpha_1 &= \frac{s\,[\eta n(\eta_2 - \eta_3) + \eta_2 n_2(\eta_3 - \eta) + \eta_3 n_3(\eta - \eta_2)]}{s_1\Delta} > 0, \\ \alpha_2 &= \frac{s\,[\eta n\,(\eta_3 - \eta_1) + \eta_1 n_1(\eta - \eta_3) + \eta_3 n_3(\eta_1 - \eta)]}{s_2\Delta} > 0, \\ \alpha_3 &= \frac{s\,[\eta n(\eta_1 - \eta_2) + \eta_1 n_1(\eta_2 - \eta) + \eta_2 n_2(\eta - \eta_1)]}{s_3\Delta} > 0. \end{aligned} \tag{40}$$

Hence, in $\mathcal{ORS}$, an odorant $(n, \eta, s)$ is a combination of

$$\begin{aligned} F_{(n_1,\eta_1,s_1)} &= \frac{F_{\max}}{1 + \left(\dfrac{1 + s_1 X}{\eta_1 s_1 X}\right)^{n_1}}, \\ F_{(n_2,\eta_2,s_2)} &= \frac{F_{\max}}{1 + \left(\dfrac{1 + s_2 X}{\eta_2 s_2 X}\right)^{n_2}}, \\ F_{(n_3,\eta_3,s_3)} &= \frac{F_{\max}}{1 + \left(\dfrac{1 + s_3 X}{\eta_3 s_3 X}\right)^{n_3}} \end{aligned}$$

by means of *positive scalars* $\alpha_1, \alpha_2, \alpha_3$,if and only if (37) e (40) are fulfilled. Due to the restriction (40), no triple can cover the whole positive octant.

In order to visualize the generation procedure in the odor response space $\mathcal{ORS}$, we first point out that, from a mathematical viewpoint, two different

odorants with the same parameters $(n, \eta)$ behave as if they were the same at different concentrations. Hence $\mathcal{ORS}$ can be effectively represented in the plane $(n, \eta)$ as its first quarter. Combinations in $\mathcal{ORS}$ corresponds to convex combinations through the map

$$(n, \eta) \mapsto (n\eta, \eta). \tag{41}$$

For instance, let us consider the basis

$$\begin{aligned}
(n_1, \eta_1, s_1): &\quad n_1 = 0.1, \quad \eta_1 = 0.1, \quad s_1 > 0 \\
(n_2, \eta_2, s_2): &\quad n_2 = 0.1, \quad \eta_2 = 18, \quad s_2 > 0 \\
(n_3, \eta_3, s_3): &\quad n_3 = 18 \quad \eta_3 = 0.1, \quad s_3 > 0.
\end{aligned} \tag{42}$$

In Fig. 1 is represented the region it generates.

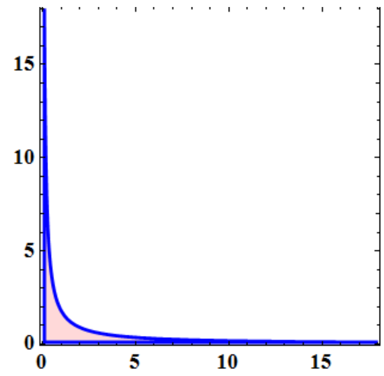


Figure 1

It is a triangle (convex hull of three points) in the space $(n\eta, \eta)$,deformed according to the map (41).

Suppose now that odorant parameters $(n, \eta, s)$ are constrained as follows

$$n \in [n_{\min}, n_{\max}], \quad \eta \in [\eta_{\min}, \eta_{\max}], \quad s > 0. \tag{43}$$

It is pretty clear that a triple can not generate all of them. However, four suitable odorants will suffice. Indeed, the map (41) tranforms lines of type $n =$const and $\eta =$const into lines in the plane $(n\eta, \eta)$. Hence the convex hull given by the four vertices

$$\begin{array}{ll}
(n_{\min}, \eta_{\min}, s_1), & s_1 > 0 \\
(n_{\min}, \eta_{\max}, s_2), & s_2 > 0 \\
(n_{\max}, \eta_{\min}, s_3), & s_3 > 0 \\
(n_{\max}, \eta_{\max}, s_4), & s_4 > 0
\end{array} \tag{44}$$

covers every odorant $F_{(n,\eta,s)}$ verifying (43).

We remark that if we consider the following two set of three vertices (44)

$$\mathcal{B}_1 = \left\{ \begin{array}{ll} (n_{\min}, \eta_{\max}, s_2), & s_2 > 0 \\ (n_{\max}, \eta_{\min}, s_3), & s_3 > 0 \\ (n_{\max}, \eta_{\max}, s_4), & s_4 > 0 \end{array} \right\}, \quad \mathcal{B}_2 = \left\{ \begin{array}{ll} (n_{\min}, \eta_{\min}, s_1), & s_1 > 0 \\ (n_{\min}, \eta_{\max}, s_2), & s_2 > 0 \\ (n_{\max}, \eta_{\min}, s_3), & s_3 > 0 \end{array} \right\},$$

each of them satisfies condition (38). Infact, since $n_{\min} < n_{\max}$, and $\eta_{\min} < \eta_{\max}$, we obtain for $\mathcal{B}_1$ and $\mathcal{B}_2$, respectively

$$\begin{aligned} \Delta_1 &\equiv \eta_{\max}\eta_{\min}\,(n_{\min} - n_{\max}) - \eta^2_{\max}\,(n_{\min} - n_{\max}) + \eta_{\min}\eta_{\max}\,(n_{\max} - n_{\max}) \\ &= \eta_{\max}\,(n_{\min} - n_{\max})\,(\eta_{\min} - \eta_{\max}) \neq 0, \end{aligned}$$

$$\begin{aligned} \Delta_2 &\equiv \eta_{\min}\eta_{\max}\,(n_{\min} - n_{\min}) - \eta_{\min}\eta_{\min}\,(n_{\min} - n_{\max}) + \eta_{\max}\eta_{\min}\,(n_{\min} - n_{\max}) \\ &= \eta_{\min}\,(n_{\min} - n_{\max})\,(-\eta_{\min} + \eta_{\max}) \neq 0 \end{aligned}$$

In this way, the set $\mathcal{B}_1$ covers every odorant $F_{(n,\eta,s)}$ verifying the following constraints

$$\eta_{\min} < \eta < \eta_{\max}, \quad \frac{\eta_{\min} n_{\max}\,(\eta_{\max} - \eta) + \eta_{\max} n_{\min}\,(\eta - \eta_{\min})}{\eta\,(\eta_{\max} - \eta_{\min})} < n < n_{\max},$$

whereas the set $\mathcal{B}_2$ covers every odorant $F_{(n,\eta,s)}$ verifying the constraints

$$\eta_{\min} < \eta < \eta_{\max}, \quad n_{\min} < n < \frac{\eta_{\min} n_{\max}\,(\eta_{\max} - \eta) + \eta_{\max} n_{\min}\,(\eta - \eta_{\min})}{\eta\,(\eta_{\max} - \eta_{\min})},$$

The curve of equation

$$\eta_{\min} < \eta < \eta_{\max}, \quad n = \frac{\eta_{\min} n_{\max}\,(\eta_{\max} - \eta) + \eta_{\max} n_{\min}\,(\eta - \eta_{\min})}{\eta\,(\eta_{\max} - \eta_{\min})}$$

can be covered by all the four odorants.

For example, if we suppose that

$$n \in [0.1, 18], \quad \eta \in [0.1, 18], \quad s > 0, \tag{45}$$

we obtain that the convex hull of vertices

$$\begin{array}{ll} (0.1, 0.1, s_1), & s_1 > 0 \\ (0.1, 18, s_2), & s_2 > 0 \\ (18, 0.1, s_3), & s_3 > 0 \\ (18, 18, s_4), & s_4 > 0 \end{array} \tag{46}$$

covers every odorant $F_{(n,\eta,s)}$ verifying (45). In fact, if we consider the following two triples

$$\begin{array}{ll} (0.1, 18, s_2), & s_2 > 0 \\ (18, 0.1, s_3), & s_3 > 0 \\ (18, 18, s_4), & s_4 > 0 \end{array}, \qquad \begin{array}{ll} (0.1, 0.1, s_1), & s_1 > 0 \\ (0.1, 18, s_2), & s_2 > 0 \\ (18, 0.1, s_3), & s_3 > 0 \end{array}$$

we easily verify that generate the regions in Figs 2 and 3, respectively.

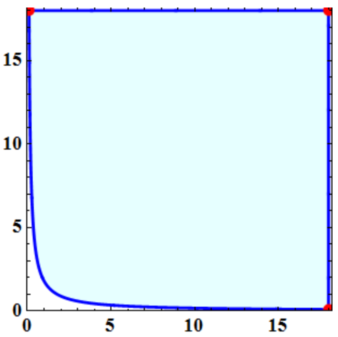


Figure 2

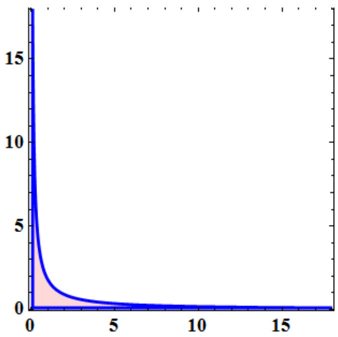


Figure 3

We remark that the union of the regions in Figs.2-3 is able to cover the entire rectangle $[0.1, 18] \times [0.1, 18]$ in the phase plane $(n, \eta)$ except for the curve of equation

$$\eta = \frac{9}{5n}, \quad 0.1 < n < 18. \tag{47}$$

Therefore, to include the curve (47) we use the four vectors (46). In particular, setting

$$s = s_1 = s_2 = s_3 = s_4 = 1,$$

we obtain that

$$\left(n, \frac{9}{5n}, 1\right) = (\alpha_1 \divideontimes (0.1, 0.1, 1)) \bullet (\alpha_2 \divideontimes (0.1, 18, 1)) \bullet (\alpha_3 \divideontimes (18, 0.1, 1)) \bullet (\alpha_4 \divideontimes (18, 18, 1))$$

in which

$$\alpha_2 = \frac{18 - n}{179n} - \frac{\alpha_1}{180}, \quad \alpha_3 = \frac{18(10n - 1)}{179n} - \alpha_1, \quad \alpha_4 = \frac{\alpha_1}{180},$$

and

$$\alpha_1 < \begin{cases} \dfrac{180n - 18}{179n}, & \text{if } \dfrac{1}{10} < n \leq \dfrac{181}{20}, \\ \dfrac{3240 - 180n}{179n}, & \text{if } \dfrac{181}{20} < n < 18. \end{cases}.$$

This shows that a point of the curve (47) (except the endpoints) can be obtained in infinitely many ways as a combination of the four vectors with positive coefficients, and in exactly one way as a combination of the two vectors $(0.1.18, 1)$, $(18, 0.1, 1)$ with positive coefficients.

## Appendix A.6

We consider a set of four OSN responses to concentration of odorants $U, V$

$$\begin{array}{ccc} \text{OSN} & \text{odorant } U & \text{odorant } V \\ 1 & n_U = 11.5, \eta_U = 4.5, K_U = 8 \times 10^{-5} & n_V = 6.7, \eta_V = 10.5, K_V = 10^{-4} \\ 2 & n_U = 13.7, \eta_U = 3.5, K_U = 8 \times 10^{-5} & n_V = 3.4, \eta_V = 11.4, K_V = 10^{-4} \\ 3 & n_U = 14.5, \eta_U = 2.8, K_U = 8 \times 10^{-5} & n_V = 4.3, \eta_V = 12.6, K_V = 10^{-4} \\ 4 & n_U = 12.5, \eta_U = 1.9, K_U = 8 \times 10^{-5} & n_V = 5.9, \eta_V = 13.9, K_V = 10^{-4} \end{array} \tag{48}$$

Owing to (15), the OSN responses to binary mixture $U + V$ for $r = 1$ are

$$\begin{array}{cc} \text{OSN} & \text{binary mixture } U + V \\ 1 & \bar{n} = 8.374, \bar{\eta} = 7.167, \bar{K} = 4.4 \times 10^{-5} \\ 2 & \bar{n} = 6.256, \bar{\eta} = 7.011, \bar{K} = 4.4 \times 10^{-5} \\ 3 & \bar{n} = 6.517, \bar{\eta} = 7.155, \bar{K} = 4.4 \times 10^{-5} \\ 4 & \bar{n} = 6.863, \bar{\eta} = 7.233, \bar{K} = 4.4 \times 10^{-5} \end{array} \tag{49}$$

Since the odorant parameters $(n, \eta, K)$ are constrained as follows

$$n \in [0.1, 18], \quad \eta \in [0.1, 18], \quad s > 0,$$

it is easy to verify that the OSN responses to odorants $U, V$ and $U + V$ are located into the region

$$\eta > \frac{9}{5n}, \quad 0.1 < n < 18,$$

of the plane $(n, \eta)$. Then, this region is generated by the basis

$$\begin{array}{ll} (0.1, 18, s_1), & s_1 > 0 \\ (18, 0.1, s_2), & s_2 > 0 \\ (18, 18, s_3), & s_3 > 0 \end{array} \tag{50}$$

and setting $s_1 = s_2 = s_3 = 10^5$, we obtain that all the OSN responses to odorants $U, V$ and $U + V$ are combination of vectors (50), i.e.

$$\left(\alpha_1 \circledast \left(0.1, 18, 10^5\right)\right) \bullet \left(\alpha_2 \circledast \left(18, 0.1, 10^5\right)\right) \bullet \left(\alpha_3 \circledast \left(18, 18, 10^5\right)\right)$$

according to the following values of $\alpha_1, \alpha_2, \alpha_3$

| OSN | scalars | odorant $U$ | odorant $V$ | mixture $U+V$ |
|---|---|---|---|---|
| | $\alpha_1$ | 0.011 | 0.037 | 0.048 |
| 1 | $\alpha_2$ | 0.094 | 0.042 | 0.136 |
| | $\alpha_3$ | 0.019 | 0.021 | 0.041 |
| | $\alpha_1$ | 0.006 | 0.052 | 0.057 |
| 2 | $\alpha_2$ | 0.101 | 0.037 | 0.138 |
| | $\alpha_3$ | 0.018 | 0.011 | 0.029 |
| | $\alpha_1$ | 0.004 | 0.053 | 0.057 |
| 3 | $\alpha_2$ | 0.106 | 0.030 | 0.136 |
| | $\alpha_3$ | 0.015 | 0.016 | 0.031 |
| | $\alpha_1$ | 0.004 | 0.052 | 0.056 |
| 4 | $\alpha_2$ | 0.112 | 0.023 | 0.135 |
| | $\alpha_3$ | 0.008 | 0.025 | 0.033 |

In the above example we considered for simplicity the mixture $U+V$ (at the same concentration) for all OSN. In general, different OSNs may lead to mixtures of $U$ and $V$ at different concentrations.

## References


[1] J.P Rospars, P Lánský, H.C Tuckwell, A Vermeulen, Coding of odor intensity in a steady-state deterministic model of an olfactory receptor neuron, J. Comp. Neurosci., 3 (1996), pp. 51–72

[2] Rospars,J.P., Lánský,P., Chaput,M. & Duchamp-Viret,P. Competitive and noncompetitive odorant interactions in the early neural coding of odorant mixtures. J. Neurosci. 28, 2659-2666 (2008).

[3] G. Cruz, G. Lowe, Neural coding of binary mixtures in a structurally related odorant pair. Sci Rep, 3, 1220 (2013).

[4] Chaput MA, El Mountassir F, Atanasova B, Thoma-Danguin T, Le Bon AM, Ferry B, Duchamp-Viret P, Interactions of odorants with olfactory receptors and receptor neurons match the perceptual dynamics observed for woody and fruity odorant mixtures. Eur J Neurosci 35:584–594 (2012).

[5] Duchamp-Viret, P., Duchamp, A. & Chaput, M.A. (2003) Single olfactory sensory neurons simultaneously integrate the components of an odour mixture. Eur. J. Neurosci., 18, 2690–2696.

[6] Lewis, C.D., 1982. Industrial and business forecasting methods: A practical guide to exponential smoothing and curve fitting. London: Butterworth Scientific.

[7] Wachowiak M, Cohen LB (2001) Representation of odorants by receptor neuron input to the mouse olfactory bulb, Neuron 32:723-735
